# 2-Chloro-*N*-[4-(dimethyl­amino)benzyl­idene]aniline

**DOI:** 10.1107/S1600536808036386

**Published:** 2008-11-13

**Authors:** Jian Li, Zu-Pei Liang, Xi-Shi Tai

**Affiliations:** a Department of Chemistry and Chemical Engineering, Weifang University, Weifang 261061, People’s Republic of China

## Abstract

In the title mol­ecule, C_15_H_15_ClN_2_, the dihedral angle between the aromatic is 64.1 (2)°.

## Related literature

For a related compound, see: You *et al.* (2004 ▶).
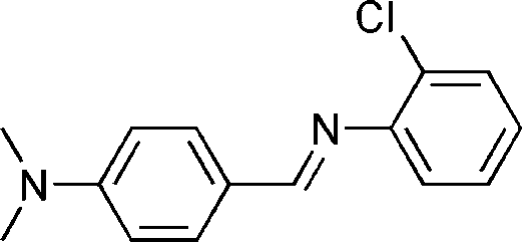


## Experimental

### 

#### Crystal data

C_15_H_15_ClN_2_
*M* *_r_* = 258.74
Orthorhombic, 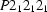

*a* = 7.7301 (8) Å
*b* = 12.2016 (18) Å
*c* = 14.047 (2) Å
*V* = 1325.0 (3) Å^3^
*Z* = 4
Mo *K*α radiation
μ = 0.27 mm^−1^
*T* = 298 (2) K
0.45 × 0.38 × 0.30 mm

#### Data collection

Bruker SMART CCD diffractometer
Absorption correction: multi-scan (*SADABS*; Bruker, 1997 ▶) *T*_min_ = 0.888, *T*_max_ = 0.923
5507 measured reflections
2318 independent reflections
1391 reflections with *I* > 2σ(*I*)
*R*_int_ = 0.054

#### Refinement

*R*[*F* ^2^ > 2σ(*F* ^2^)] = 0.045
*wR*(*F* ^2^) = 0.105
*S* = 1.02
2318 reflections
165 parameters
H-atom parameters constrained
Δρ_max_ = 0.17 e Å^−3^
Δρ_min_ = −0.18 e Å^−3^
Absolute structure: Flack (1983 ▶), 1358 Friedel pairs
Flack parameter: −0.07 (10)

### 

Data collection: *SMART* (Bruker, 1997 ▶); cell refinement: *SAINT* (Bruker, 1997 ▶); data reduction: *SAINT*; program(s) used to solve structure: *SHELXS97* (Sheldrick, 2008 ▶); program(s) used to refine structure: *SHELXL97* (Sheldrick, 2008 ▶); molecular graphics: *SHELXTL* (Sheldrick, 2008 ▶); software used to prepare material for publication: *SHELXTL*.

## Supplementary Material

Crystal structure: contains datablocks global, I. DOI: 10.1107/S1600536808036386/lh2690sup1.cif

Structure factors: contains datablocks I. DOI: 10.1107/S1600536808036386/lh2690Isup2.hkl

Additional supplementary materials:  crystallographic information; 3D view; checkCIF report

## Figures and Tables

**Fig. 1.**
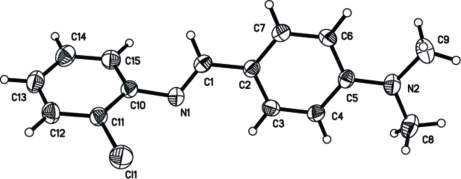
The molecular structure of (I), drawn with 30% probability ellipsoids.

## supplementary crystallographic information

### Comment

Schiff base compounds have been used as fine chemicals and medical substrates and they are important ligands in coordination chemistry due to their ease of preparation and their ability to be modified both electronically and sterically. In this paper, the structure of the title compound, (I), is reported. The molecular structure of (I) is illustrated in Fig. 1. The bond lengths and angles in the title  molecule are similar to the related compound 4-chloro-*N*-[4-(dimethylamino)benzylidene]aniline (You *et al.*, 2004). The 4-(Dimethylamino)benzylidene system is nearly planar to within 0.035 (3) A°. 2-Chlorobenzenamine system is nearly planar to within 0.060 (3) A°. The dihedral angle between these two systems is 67.0 (2) °.

### Experimental

A mixture of 4-(dimethylamino)benzaldehyde (0.01 mol) and 2-chlorobenzenamine (0.01 mol) in ethanol (10 ml) was refluxed for 2 h. After cooling, filtration and drying, the title compound was obtained. 10 mg of (I) were dissolved in 15 ml of ethanol, and the solution was kept at room temperature for 5 d. Natural evaporation gave light yellow single crystals of the title compound, suitable for X-ray analysis.

### Refinement

H atoms were initially located from difference maps and then refined in a riding model with C—H = 0.93–0.96 Å and *U*_iso_(H) = 1.2*U*_eq_(C) or 1.5*U*_eq_(methyl C).

### Crystal data

**Table:**

C_15_H_15_ClN_2_	*F*_000_ = 544
*M**_r_* = 258.74	*D*_x_ = 1.297 Mg m^−^^3^
Orthorhombic, *P*2_1_2_1_2_1_	Mo *K*α radiation λ = 0.71073 Å
Hall symbol: P 2ac 2ab	Cell parameters from 1182 reflections
*a* = 7.7301 (8) Å	θ = 2.9–20.1º
*b* = 12.2016 (18) Å	µ = 0.27 mm^−^^1^
*c* = 14.047 (2) Å	*T* = 298 (2) K
*V* = 1325.0 (3) Å^3^	Block, light yellow
*Z* = 4	0.45 × 0.38 × 0.30 mm

### Data collection

**Table:**

Bruker SMART CCD diffractometer	2318 independent reflections
Radiation source: fine-focus sealed tube	1391 reflections with *I* > 2σ(*I*)
Monochromator: graphite	*R*_int_ = 0.054
*T* = 298(2) K	θ_max_ = 25.0º
φ and ω scans	θ_min_ = 2.2º
Absorption correction: multi-scan(SADABS; Bruker, 1997)	*h* = −9→9
*T*_min_ = 0.888, *T*_max_ = 0.923	*k* = −14→13
5507 measured reflections	*l* = −9→16

### Refinement

**Table:**

Refinement on *F*^2^	Hydrogen site location: inferred from neighbouring sites
Least-squares matrix: full	H-atom parameters constrained
*R*[*F*^2^ > 2σ(*F*^2^)] = 0.045	*w* = 1/[σ^2^(*F*_o_^2^) + (0.0377*P*)^2^] where *P* = (*F*_o_^2^ + 2*F*_c_^2^)/3
*wR*(*F*^2^) = 0.105	(Δ/σ)_max_ < 0.001
*S* = 1.02	Δρ_max_ = 0.18 e Å^−^^3^
2318 reflections	Δρ_min_ = −0.18 e Å^−^^3^
165 parameters	Extinction correction: none
Primary atom site location: structure-invariant direct methods	Absolute structure: Flack (1983), 1358 Friedel pairs
Secondary atom site location: difference Fourier map	Flack parameter: −0.07 (10)

### Special details

**Table:**

Geometry. All e.s.d.'s (except the e.s.d. in the dihedral angle between two l.s. planes) are estimated using the full covariance matrix. The cell e.s.d.'s are taken into account individually in the estimation of e.s.d.'s in distances, angles and torsion angles; correlations between e.s.d.'s in cell parameters are only used when they are defined by crystal symmetry. An approximate (isotropic) treatment of cell e.s.d.'s is used for estimating e.s.d.'s involving l.s. planes.
Refinement. Refinement of *F*^2^ against ALL reflections. The weighted *R*-factor *wR* and goodness of fit *S* are based on *F*^2^, conventional *R*-factors *R* are based on *F*, with *F* set to zero for negative *F*^2^. The threshold expression of *F*^2^ > σ(*F*^2^) is used only for calculating *R*-factors(gt) *etc*. and is not relevant to the choice of reflections for refinement. *R*-factors based on *F*^2^ are statistically about twice as large as those based on *F*, and *R*- factors based on ALL data will be even larger.

### Fractional atomic coordinates and isotropic or equivalent isotropic displacement parameters (Å^2^)

**Table:**

	*x*	*y*	*z*	*U*_iso_*/*U*_eq_	
Cl1	1.22788 (14)	1.11816 (8)	−0.13149 (7)	0.0753 (4)	
N1	0.9918 (4)	0.9322 (2)	−0.09661 (18)	0.0513 (8)	
N2	0.8770 (4)	0.7449 (2)	0.32629 (18)	0.0555 (8)	
C1	1.0238 (4)	0.8391 (3)	−0.0595 (2)	0.0477 (9)	
H1	1.0788	0.7861	−0.0962	0.057*	
C2	0.9776 (4)	0.8127 (2)	0.0381 (2)	0.0436 (9)	
C3	0.8837 (4)	0.8839 (3)	0.0949 (2)	0.0460 (9)	
H3	0.8439	0.9491	0.0686	0.055*	
C4	0.8472 (5)	0.8618 (3)	0.1884 (2)	0.0478 (9)	
H4	0.7815	0.9112	0.2235	0.057*	
C5	0.9077 (4)	0.7656 (3)	0.2319 (2)	0.0441 (9)	
C6	0.9993 (5)	0.6920 (3)	0.1744 (2)	0.0498 (9)	
H6	1.0379	0.6261	0.2002	0.060*	
C7	1.0331 (5)	0.7156 (3)	0.0807 (2)	0.0510 (10)	
H7	1.0949	0.6652	0.0445	0.061*	
C8	0.7854 (5)	0.8221 (3)	0.3849 (2)	0.0719 (12)	
H8A	0.8528	0.8878	0.3913	0.108*	
H8B	0.7660	0.7908	0.4467	0.108*	
H8C	0.6763	0.8394	0.3559	0.108*	
C9	0.9648 (5)	0.6548 (3)	0.3736 (2)	0.0671 (11)	
H9A	0.9342	0.5870	0.3432	0.101*	
H9B	0.9305	0.6524	0.4393	0.101*	
H9C	1.0876	0.6654	0.3696	0.101*	
C10	1.0315 (5)	0.9502 (3)	−0.1928 (2)	0.0452 (9)	
C11	1.1315 (4)	1.0405 (3)	−0.2197 (2)	0.0481 (9)	
C12	1.1594 (5)	1.0664 (3)	−0.3137 (2)	0.0595 (10)	
H12	1.2266	1.1269	−0.3298	0.071*	
C13	1.0873 (5)	1.0020 (3)	−0.3841 (3)	0.0654 (11)	
H13	1.1042	1.0197	−0.4478	0.078*	
C14	0.9911 (5)	0.9125 (3)	−0.3600 (3)	0.0661 (11)	
H14	0.9451	0.8682	−0.4075	0.079*	
C15	0.9612 (5)	0.8869 (3)	−0.2650 (2)	0.0587 (10)	
H15	0.8932	0.8266	−0.2497	0.070*	

### Atomic displacement parameters (Å^2^)

**Table:**

	*U*^11^	*U*^22^	*U*^33^	*U*^12^	*U*^13^	*U*^23^
Cl1	0.0818 (8)	0.0730 (7)	0.0711 (6)	−0.0204 (6)	−0.0003 (6)	−0.0069 (5)
N1	0.060 (2)	0.0516 (17)	0.0423 (16)	−0.0012 (18)	0.0060 (16)	0.0035 (14)
N2	0.053 (2)	0.069 (2)	0.0440 (17)	0.0048 (18)	0.0025 (15)	0.0064 (15)
C1	0.048 (3)	0.050 (2)	0.045 (2)	−0.0014 (19)	−0.0003 (19)	−0.0074 (17)
C2	0.046 (2)	0.0450 (19)	0.0397 (19)	−0.0033 (19)	0.0012 (18)	−0.0035 (16)
C3	0.049 (2)	0.0394 (18)	0.049 (2)	0.0003 (19)	−0.0047 (17)	0.0001 (18)
C4	0.050 (2)	0.048 (2)	0.045 (2)	0.0075 (17)	0.0021 (18)	−0.0034 (17)
C5	0.042 (2)	0.052 (2)	0.0386 (19)	−0.0057 (18)	−0.0011 (17)	−0.0029 (18)
C6	0.057 (3)	0.0407 (19)	0.051 (2)	0.005 (2)	−0.003 (2)	0.0055 (17)
C7	0.056 (3)	0.049 (2)	0.048 (2)	0.0044 (19)	0.0023 (19)	−0.0053 (18)
C8	0.076 (3)	0.095 (3)	0.045 (2)	0.004 (3)	0.012 (2)	−0.002 (2)
C9	0.062 (3)	0.078 (3)	0.061 (2)	−0.007 (2)	−0.004 (2)	0.024 (2)
C10	0.047 (2)	0.046 (2)	0.043 (2)	0.0044 (19)	0.0025 (19)	0.0001 (17)
C11	0.047 (2)	0.050 (2)	0.047 (2)	0.0046 (19)	0.0041 (19)	0.0020 (17)
C12	0.058 (3)	0.063 (2)	0.058 (2)	−0.002 (2)	0.014 (2)	0.009 (2)
C13	0.069 (3)	0.081 (3)	0.046 (2)	0.019 (2)	0.008 (2)	0.008 (2)
C14	0.068 (3)	0.080 (3)	0.050 (2)	0.007 (3)	−0.006 (2)	−0.007 (2)
C15	0.062 (3)	0.057 (2)	0.057 (2)	−0.003 (2)	0.000 (2)	−0.001 (2)

### Geometric parameters (Å, °)

**Table:**

Cl1—C11	1.728 (3)	C7—H7	0.9300
N1—C1	1.274 (3)	C8—H8A	0.9600
N1—C10	1.403 (4)	C8—H8B	0.9600
N2—C5	1.371 (4)	C8—H8C	0.9600
N2—C8	1.438 (4)	C9—H9A	0.9600
N2—C9	1.453 (4)	C9—H9B	0.9600
C1—C2	1.453 (4)	C9—H9C	0.9600
C1—H1	0.9300	C10—C15	1.385 (4)
C2—C3	1.385 (4)	C10—C11	1.398 (4)
C2—C7	1.395 (4)	C11—C12	1.376 (4)
C3—C4	1.369 (4)	C12—C13	1.379 (5)
C3—H3	0.9300	C12—H12	0.9300
C4—C5	1.404 (4)	C13—C14	1.364 (5)
C4—H4	0.9300	C13—H13	0.9300
C5—C6	1.400 (4)	C14—C15	1.390 (4)
C6—C7	1.373 (4)	C14—H14	0.9300
C6—H6	0.9300	C15—H15	0.9300
			
?···?	?		
			
C1—N1—C10	119.4 (3)	N2—C8—H8C	109.5
C5—N2—C8	121.2 (3)	H8A—C8—H8C	109.5
C5—N2—C9	120.1 (3)	H8B—C8—H8C	109.5
C8—N2—C9	117.6 (3)	N2—C9—H9A	109.5
N1—C1—C2	122.5 (3)	N2—C9—H9B	109.5
N1—C1—H1	118.8	H9A—C9—H9B	109.5
C2—C1—H1	118.8	N2—C9—H9C	109.5
C3—C2—C7	116.5 (3)	H9A—C9—H9C	109.5
C3—C2—C1	122.2 (3)	H9B—C9—H9C	109.5
C7—C2—C1	121.2 (3)	C15—C10—C11	117.3 (3)
C4—C3—C2	122.5 (3)	C15—C10—N1	122.2 (3)
C4—C3—H3	118.7	C11—C10—N1	120.2 (3)
C2—C3—H3	118.7	C12—C11—C10	121.8 (3)
C3—C4—C5	120.9 (3)	C12—C11—Cl1	119.7 (3)
C3—C4—H4	119.6	C10—C11—Cl1	118.5 (3)
C5—C4—H4	119.6	C11—C12—C13	119.6 (3)
N2—C5—C6	121.8 (3)	C11—C12—H12	120.2
N2—C5—C4	121.2 (3)	C13—C12—H12	120.2
C6—C5—C4	117.0 (3)	C14—C13—C12	119.9 (3)
C7—C6—C5	121.0 (3)	C14—C13—H13	120.0
C7—C6—H6	119.5	C12—C13—H13	120.0
C5—C6—H6	119.5	C13—C14—C15	120.6 (4)
C6—C7—C2	122.1 (3)	C13—C14—H14	119.7
C6—C7—H7	119.0	C15—C14—H14	119.7
C2—C7—H7	119.0	C10—C15—C14	120.8 (3)
N2—C8—H8A	109.5	C10—C15—H15	119.6
N2—C8—H8B	109.5	C14—C15—H15	119.6
H8A—C8—H8B	109.5		
			
C10—N1—C1—C2	−176.5 (3)	C3—C2—C7—C6	−1.0 (5)
N1—C1—C2—C3	5.2 (5)	C1—C2—C7—C6	176.2 (3)
N1—C1—C2—C7	−171.9 (3)	C1—N1—C10—C15	58.8 (5)
C7—C2—C3—C4	0.5 (5)	C1—N1—C10—C11	−127.5 (4)
C1—C2—C3—C4	−176.7 (3)	C15—C10—C11—C12	0.1 (5)
C2—C3—C4—C5	1.4 (5)	N1—C10—C11—C12	−173.9 (3)
C8—N2—C5—C6	178.9 (3)	C15—C10—C11—Cl1	−177.6 (2)
C9—N2—C5—C6	10.8 (5)	N1—C10—C11—Cl1	8.4 (4)
C8—N2—C5—C4	−1.6 (5)	C10—C11—C12—C13	0.1 (5)
C9—N2—C5—C4	−169.6 (3)	Cl1—C11—C12—C13	177.7 (3)
C3—C4—C5—N2	177.6 (3)	C11—C12—C13—C14	−1.0 (6)
C3—C4—C5—C6	−2.8 (5)	C12—C13—C14—C15	1.6 (6)
N2—C5—C6—C7	−178.1 (3)	C11—C10—C15—C14	0.6 (5)
C4—C5—C6—C7	2.3 (5)	N1—C10—C15—C14	174.4 (3)
C5—C6—C7—C2	−0.4 (5)	C13—C14—C15—C10	−1.5 (6)

### Hydrogen-bond geometry (Å, °)

**Table:**

*D*—H···*A*	*D*—H	H···*A*	*D*···*A*	*D*—H···*A*
?—?···?	?	?	?	?
